# Methods for detecting, building, and improving tryptophan mannosylation in glycoprotein structures

**DOI:** 10.1002/pro.70025

**Published:** 2025-01-22

**Authors:** Lou Holland, Phuong Thao Pham, Haroldas Bagdonas, Jordan S. Dialpuri, Lucy C. Schofield, Jon Agirre

**Affiliations:** ^1^ York Structural Biology Laboratory, Department of Chemistry University of York York UK

**Keywords:** carbohydrates, *C*‐glycans, model building, validation

## Abstract

Tryptophan mannosylation, the covalent addition of an α‐ᴅ‐mannose sugar to a tryptophan side chain, is a post‐translational modification (PTM) that can affect protein stability, folding, and interactions. Compared to other forms of protein glycosylation, it is relatively uncommon but is affected by conformational anomalies and modeling errors similar to those seen in *N*‐ and *O*‐glycans in the Protein Data Bank (PDB). In this work, we report methods for detecting, building, and improving mannose structures linked to tryptophans. These methods have been used to mine X‐ray crystallographic and cryo‐electron microscopy maps in the PDB looking for unmodeled mannosylation, resulting in a number of cases where the modification can be placed in the map with high confidence. Additionally, we address most conformational issues affecting this modification. Finally, the development of a structural template to recognize thrombospondin repeats (TSR) domains where tryptophan mannosylation occurs will allow for the mannosylation of candidate‐predicted models, for example, those predicted with AlphaFold.

## 1 INTRODUCTION

Accurate modeling of protein glycosylation is still a developing field, despite glycans being one of the most common post‐translational modifications (PTMs) (Frank et al., 2020; Schofield et al., 2024; Spiro, 2002). Whilst the task of accurately building the more common *N*‐glycans and *O*‐glycans remains a challenge, they are at least well‐known. *C*‐glycans; however, are perhaps less well‐known, owing to their more recent discovery (Hofsteenge et al., 1994).

**FIGURE 1:**
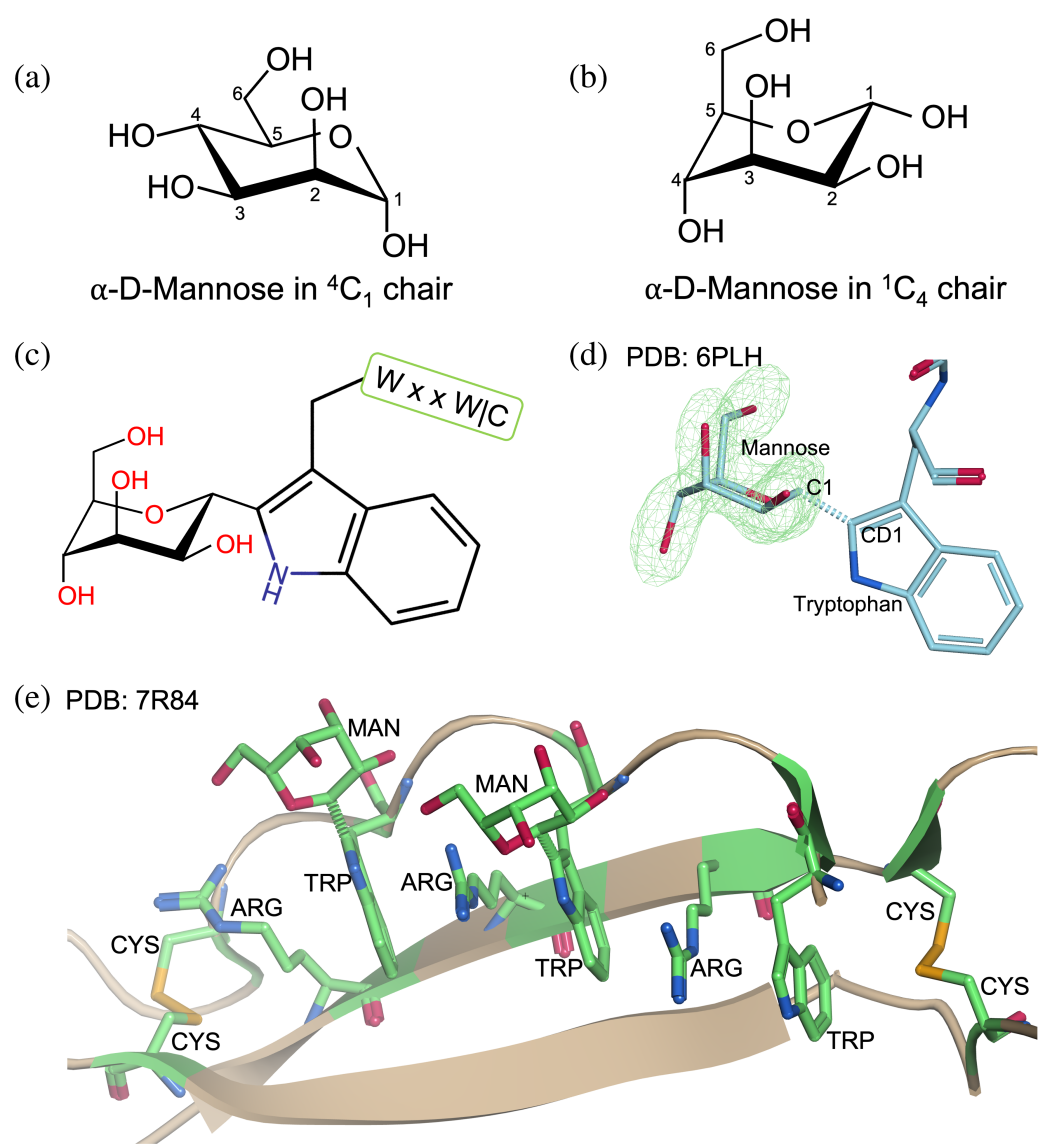
An overview of *C*‐mannosylation. Panels (a, b) show the difference between the ^4^C_1_ and ^1^C_4_ chair conformations for α‐ᴅ‐mannose, with the ^1^C_4_ chair being the lowest energy conformation for this PTM. Panel (c) shows the PTM in a 3D line drawing and (d) shows an example of this PTM currently in the PDB from the structure with PDB accession code 6PLH (John et al., 2021) with an omit map for the mannose residue at 5*σ*. A glycosidic linkage is formed between the C1 atom of an α‐ᴅ‐mannose in a ^1^C_4_ chair and the CD1 atom of a tryptophan which sits within the consensus sequence WXXW|C. Often, this consensus sequence sits within a thrombospondin‐repeat (TSR) domain as shown in panel (e), taken from the structure with PDB accession code 7R84 (Wang et al., 2022), where there are three antiparallel strands, and tryptophans are stacked by arginines as a ladder, capped by two disulphide bonds shown in orange. The residues which form this *C*‐mannosylation consensus sequence are shown in green in the bond representation whereas the rest of the protein is shown in beige in the ribbon representation. Panel (c) was produced from PDB Chemical Sketch Tool (https://www.rcsb.org/chemical‐sketch) and panels (d) and (e) were drawn by https://moorhen. org /.

Unlike *N*‐glycans and *O*‐glycans, which exist in multiple forms, there is only one known type of *C*‐glycosylation, known as tryptophan‐mannosylation or *C*‐mannosylation. *C*‐mannosylation describes a protein modification in which a rare carbon–carbon glycosidic bond is formed between the indole C2 atom of a tryptophan residue (labeled CD1 from this point onwards) and the anomeric C1 atom of an α‐ᴅ‐mannose sugar (de Beer et al., 1995; Hofsteenge et al., 1994). This sugar is preferably in the ^1^C_4_ conformation rather than the more usual ^4^C_1_ conformation (de Beer et al., 1995). The difference between these chair conformations is illustrated in Figure 1a,b, and visual representations of the modification are shown in Figure 1c,d (John et al., 2021). This modification occurs in the endoplasmic reticulum of metazoan and apicomplexan protist cells (Doucey et al., 1998; John et al., 2021; Krieg et al., 1997), where C‐mannosylation enzyme (C‐mannosyltransferase) co‐translationally modifies a tryptophan in the consensus sequence WxxW|C (Bloch et al., 2023; Buettner et al., 2013; Krieg et al., 1998). However, not every tryptophan found in the relevant consensus sequence will be mannosylated.

Presently, *C*‐mannosylation has been commonly found in proteins with Thrombospondin repeat (TSR) domains (such as Thrombospondin‐1, properdin, terminal complement components, etc.) and type I cytokine receptors (Hofsteenge et al., 1999). The consensus sequence in these proteins primarily falls within a Thrombospondin repeat domain (Pfam entry PF00090) (Bateman et al., 2004; Hofsteenge et al., 1999; John et al., 2021). The Thrombospondin repeat domain, as shown in Figure 1e, has two regular β‐strands and one rippled strand, which contains the double consensus sequences WxxWxxW|C (Tan et al., 2002). Three tryptophans on the rippled strand are intercalated by three arginines from the next strand and capped by two conserved disulfide bonds (Crine & Acharya, 2022). The double consensus sequence can have one, two, or three *C*‐mannosylated tryptophans (Hofsteenge et al., 1999; John et al., 2021), a phenomenon not dissimilar to the microheterogeneity of other forms of protein glycosylation. By contrast, the TSR domain of type I cytokine receptors has only the single consensus sequence, in which *C*‐mannosylation happens on the first tryptophan residue, and does not have the conserved disulfide bonds (Crine & Acharya, 2022).

It is important to improve our understanding and modeling of *C*‐mannosylation, as it is believed to impact the overall protein structure and properties: *C*‐mannosylation supports protein folding (Doucey et al., 1998), stabilizes protein domains (Shcherbakova et al., 2019), increases protein solubility (Hartmann & Hofsteenge, 2000), and regulates protein–protein interactions (Hamming et al., 2012; Pronker et al., 2016). The function of *C*‐mannosylation is the subject of current research and debate (Frank et al., 2020), and before this study, there were no specific pieces of software that helped with modeling *C*‐mannosylation correctly.

In some ways, the singular nature of *C*‐mannosylation makes it simpler to model than *N*‐glycans and *O*‐glycans. If electron density supports a modification approximately the size of a pyranose ring adjacent to the CD1 atom of a tryptophan residue within the consensus sequence, α‐ᴅ‐mannose is the only sugar to be modeled. However, for lower‐resolution 3D models, without appropriate restraints on the torsion angles in the sugar ring, refinement can distort the mannose residue into a high‐energy conformation and skew the carbon–carbon glycosidic bond. Consequently, the mannose moiety is frequently modeled incorrectly with high‐energy ring conformations in many deposited glycoprotein structures (Frank et al., 2020). It is possible that this is, in part, due to many people being more familiar with *N*‐glycans, and expecting *C*‐glycans to follow the same pattern. ᴅ‐pyranosides in *N*‐glycans typically assume the stable ^4^C_1_ conformation; however, *C*‐mannose favorably adopts the inverse conformation, ^1^C_4_. This is because the ^1^C_4_ conformation reduces the steric clashes between the hydroxymethyl group of the glycan and the indole ring of tryptophan (de Beer et al., 1995). The difference between these chair conformations is illustrated in Figure 1a,b.

The study of this modification would be aided by an increase in the number of available 3D models containing *C*‐mannosylated tryptophans. There are currently only 43 entries in the Protein Data Bank (PDB) (Berman et al., 2000) with *C*‐mannosylated tryptophans present compared to approximately 12,000 entries containing *N*‐glycans (Dialpuri, Bagdonas, Schofield, Pham, Holland, & Agirre, 2024; Schofield et al., 2024). Previous investigations identified more than 12,000 protein sequences which have the consensus sequence WxxW (Krieg et al., 1998). A more recent study into predicting *C*‐mannosylation using elements of the protein geometry around the consensus sequence such as secondary structure elements and surface accessibility estimated nearly 2600 potential *C‐*mannosylated proteins in the human proteome (Julenius, 2007). While it is unlikely that there are thousands of missing *C*‐mannosylations in the PDB, it suggests the potential for a number of entries with unmodeled mannosylated tryptophans.

The main aim of this work is to provide methods that can be used to improve *C*‐glycan model quality in the PDB for both already deposited 3D models and models that will be deposited in the future. This can be further broken down into two sub‐aims. The first is to improve *C*‐glycan models already present in the PDB which may have been incorrectly modeled or distorted by refinement (Agirre, Davies, et al., 2015), and the second is to identify and correctly model unmodeled *C*‐glycans. The methods presented here have also been extended with a structural fingerprint of the TSR domain, which may be used post‐prediction (Bagdonas et al., 2021) to mannosylate candidate regions in predicted models such as those calculated by AlphaFold (Abramson et al., 2024).

## 2 RESULTS AND DISCUSSION

The protocol developed was run on a mirror of the PDB (Berman et al., 2000) downloaded on the July 25, 2024, first to identify and model unmodeled *C*‐mannosylation, and second to identify mismodeled *C*‐mannosylation and remodel them accurately.

### 2.1 Identifying and modeling unmodelled *C*‐glycans in the PDB

Ten unmodelled *C*‐mannosylation sites were identified across five X‐ray crystallography structures to a high degree of confidence, meaning that the PTM was well supported by the electron density when checked visually, as well as having a real space correlation coefficient (RSCC) above 0.6. These models were PDB entries with accession codes 4V2A (Seiradake et al., 2014), 5FTT (Jackson et al., 2016), 7NOZ (Lorentzen et al., 2022), 7ZA2 (Akkermans et al., 2022), and 7ZA3 (Akkermans et al., 2022).

An additional 16 unmodeled *C*‐mannosylation sites were identified to a high degree of confidence across three related Cryo‐EM structures: PDB 8B0F, 8B0G, and 8B0H (Couves et al., 2023). Additional sites in these models also showed potential unmodeled *C*‐mannosylation, but were deemed to be of lower confidence due to the mannose residues modeled there having low RSCC values.

A summary of the high‐confidence results for both X‐ray and Cryo‐EM structures is given in Table A1.

Of the structures in which unmodeled *C*‐mannosylation was identified with high confidence, three already had *C*‐mannosylation modeled at different tryptophan residues, and five had no *C*‐mannosylation at all. The three models which already contained *C*‐mannosylation had PDB accession codes 7NOZ, 7ZA2, and 7ZA3. The remaining structures which did not contain any *C*‐mannosylation prior to this work had PDB accession codes 4V2A, 5FTT, 8B0F, 8B0G, and 8B0H.

The UniProt IDs (UniProt Consortium, 2023) corresponding to the chains where unmodeled *C*‐mannosylation sites were identified were checked to see if they contained a PTM annotation corresponding to *C*‐mannosylation. These UniProt IDs can be found in the results summary in Table A1. In the majority of the protein chains where unmodelled *C*‐mannosylation sites were identified, corresponding to UniProt IDs Q6ZN44, P27918, P01031, P07358, and P07357, there was an existing PTM annotation for *C*‐mannosylation. However, no such annotation existed for UniProt ID F1LW30, which corresponded to six of the protein chains identified as having previously unmodeled *C*‐mannosylation across models with PDB accession codes 5FTT, 7ZA2, and 7ZA3.

**FIGURE 2:**
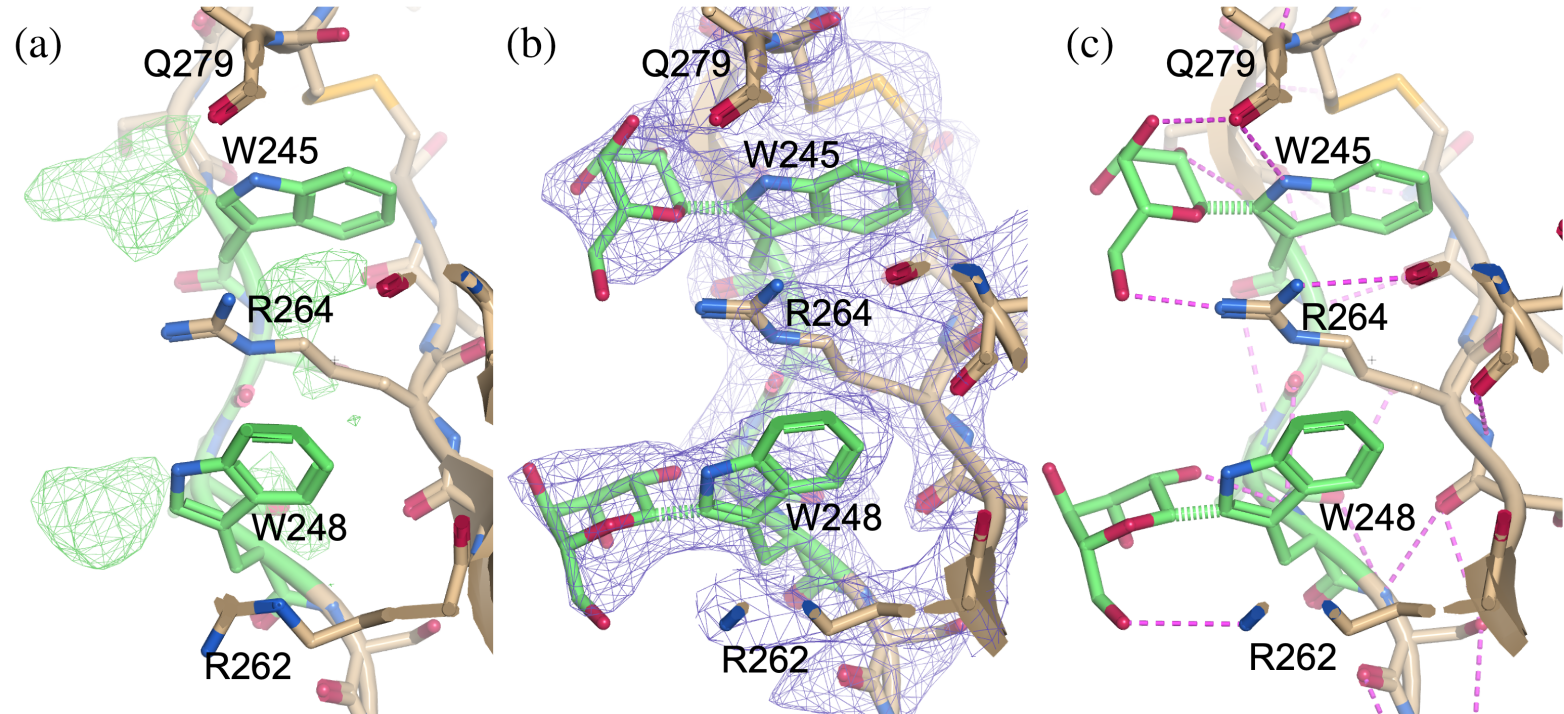
Chain A from glycoprotein structure with PDB accession code 4V2A, focused on Trp‐245 and Trp‐248 (a) before and (b) after modeling *C*‐mannosylation. The difference density map contour is at 3*σ* and the electron density map contour is at 1*σ*. Panel (c) shows hydrogen bonds as pink dashed lines between the mannose residues and side chains Arg‐262, Arg‐264, and Gln‐279 on the protein backbone as well as the Trp residues they are covalently bonded to. All possible hydrogen bonds were suggested by *Moorhen* (regardless of the hydrogen's orientation), and are likely overestimated. The initial 3D model was taken from PDB 4V2A (Seiradake et al., 2014) and the figure was produced using https://moorhen.org/.

An example from a human Unc5A ectodomain in the X‐ray crystal structure with PDB accession code 4V2A (Seiradake et al., 2014) is shown in Figure 2, where the identified *C*‐mannosylation sites were in the typical TSR domain. Figure 2a shows the initial 3D model along with the difference density map at 3*σ*. Two blobs of positive difference density can be seen close to Trp‐245 and Trp‐248 in chain A of the protein structure. Figure 2b shows the 3D model after the *C*‐mannosylation was modeled, with two mannose residues now occupying those positions, fitting well into the electron density map at 1*σ*. Analyzing the possible contacts between the glycans and the protein neighbors showed that the two mannose residues could potentially form multiple intramolecular hydrogen bonds within the TSR domain. Figure 2c shows possible hydrogen bonds that could form between the mannose residues and the rest of the local structure, improving the stability of the TSR domain.

**FIGURE 3:**
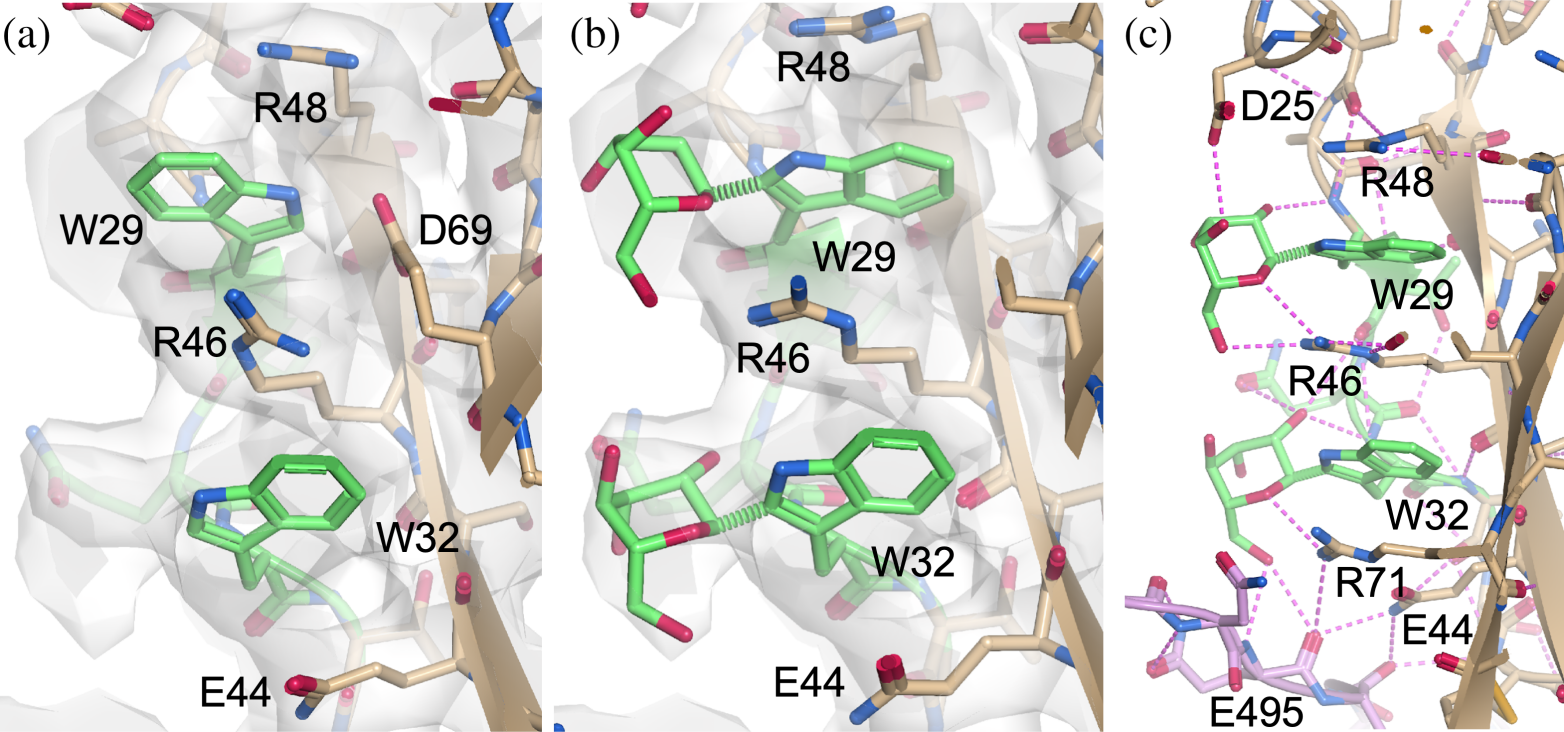
Chain B from the glycoprotein structure with PDB accession code 8B0F (Couves et al., 2023), focused on Trp‐29 and Trp‐32 (a) before and (b) after modeling *C*‐mannosylation. (c) Hydrogen‐bond network (pink dash lines) of the two mannose residues. The two C‐glycans form hydrogen bonds with the protein residues in the TSR domain of the same chain (beige) and also with those from another chain (purple) in the protein complex. All possible hydrogen bonds were suggested by *Moorhen* (regardless of the hydrogen's orientation), and are likely overestimated.

Figure 3 shows an example of an unmodeled *C‐*mannosylation found in a membrane attack complex with inhibitor CD59 in the Cryo‐EM structure with PDB accession code 8B0F (Couves et al., 2023). In this model, one of the tryptophan residues identified as a potential *C*‐mannosylation site, TRP‐29 in chain B, was found to be in the wrong orientation, preventing a mannose residue from fitting in the correct position without producing clashes. Therefore, prior to modeling the mannose residue, the tryptophan was flipped manually in Coot (Emsley & Cowtan, 2004). The two newly modeled glycans shown in Figure 3 were found to have an RSCC of 0.64 and 0.60, and *Q*‐scores of 0.63 and 0.59, respectively. The RSCC and *Q*‐scores, consistent with model observing, indicated that the modeled *C*‐mannose was fairly fitted in the EM map.

As with the X‐ray structure shown in Figure 2, the mannosylated tryptophans were in the typical TSR domain, as is commonly observed with *C‐*mannosylation. Multiple possible inter‐ and intra‐molecular hydrogen bonds were observed between the mannoses and the neighboring residues, shown in Figure 3c. It therefore appears that the *C*‐mannosylation could aid in directing the folding of TSR domains, as has been suggested in previous studies (Shcherbakova et al., 2019), as well as stabilizing the protein complex.

### 2.2 Fixing distorted *C*‐glycans in the PDB

Forty‐five problematic *C*‐glycans were identified across 18 X‐ray crystallography structures, along with 45 problematic *C*‐glycans across six Cryo‐EM structures. These were replaced with a mannose residue in the correct conformation before being refined, using restraints generated by *Privateer* (Agirre, Iglesias‐Fernández, et al., 2015), to maintain the ring conformation and carbon–carbon glycosidic bond geometry. The RSCC of the residue was calculated before and after this process; it was expected that the RSCC of the initial glycan would likely be higher than the corrected version, as when unrestrained, the mannose residue can be skewed to fit the electron density. However, in many cases, the process of fixing the conformation and linkage also improved the RSCC. Finally, during this process, a number of *C*‐mannosylation sites were identified to be poorly supported by the electron density; therefore, the mannose residues were removed. The results of this process were then manually checked, and are summarized in Tables A2 and A3 in the appendix. Table A2 contains the results for X‐ray crystal structures, while Table A3 contains the results for Cryo‐EM structures.

**FIGURE 4:**
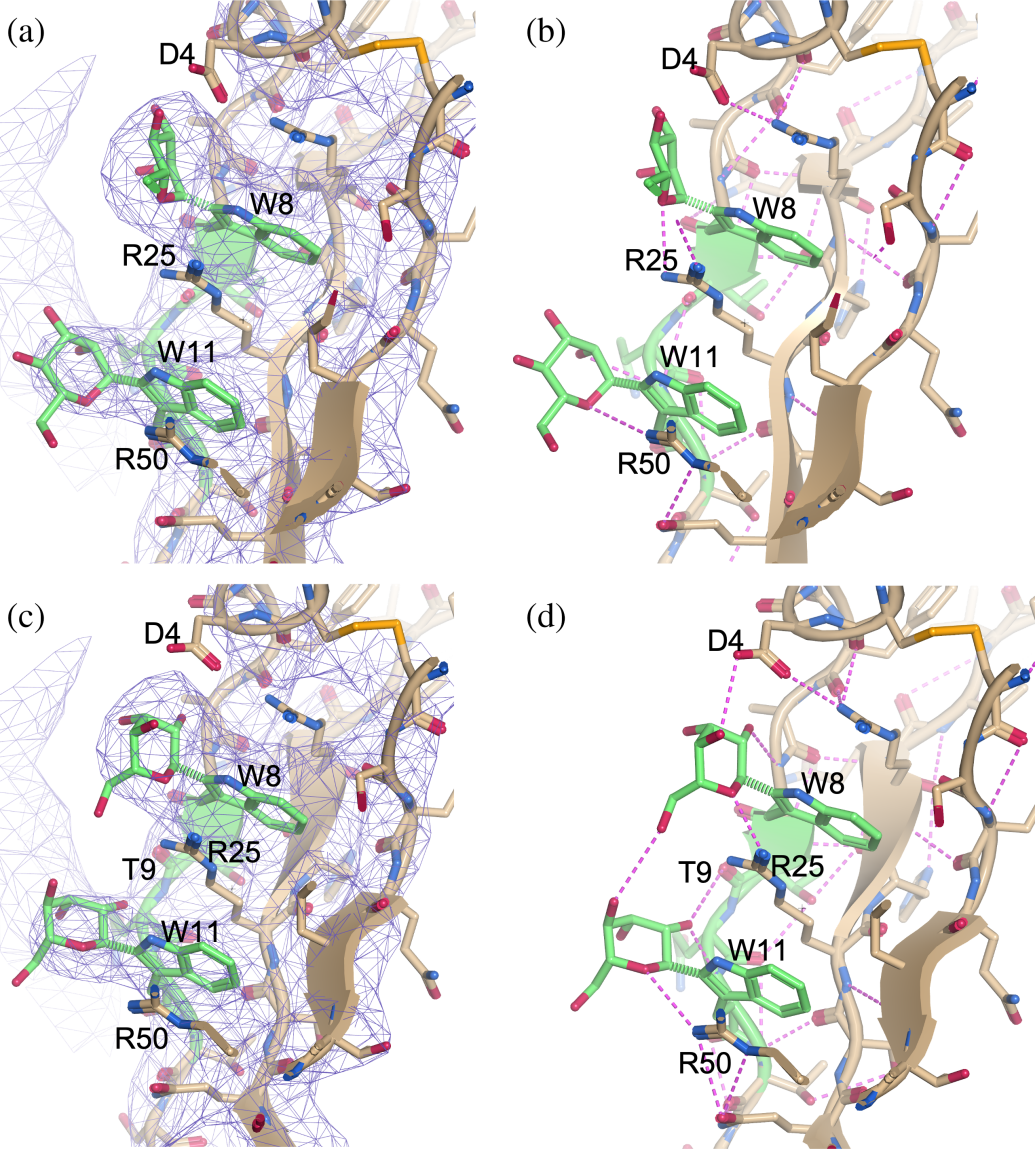
An example result of a corrected *C‐*mannosylation site, showing structure 4E0S chain B, Trp‐8 and Trp‐11. Panel (a, b) show the structure before fixing and panels (c, d) show it after fixing. In both cases, the map contour is set at 1*σ* and pink dashed lines show all possible hydrogen bonds as suggested by *Moorhen*. Before fixing, *Moorhen* suggests possible hydrogen bonds between the mannose residues and Asp‐4, Trp‐11, Arg‐25, and Arg‐50. After fixing, Moorhen suggests possible hydrogen bonds between the mannose residues and Asp‐4, Trp‐8, Thr‐9, Trp‐11, Arg‐25, and Arg‐50. The initial 3D model was taken from PDB 4E0S (Aleshin et al., 2012) and the figure was produced by https://moorhen.org/.

An example is shown in Figure 4, taken from the X‐ray crystal structure of C5b‐6 with PDB accession code 4E0S (Aleshin et al., 2012). In the original 3D model, the mannose residues covalently bonded to Trp‐8 and Trp‐11 in chain B were in the higher energy ^4^C_1_ chair conformation. These were replaced with mannose residues in the appropriate ^1^C_4_ chair conformation. It can be seen that these fixed models still fit well within the density map at 1*σ*. In addition, fixing the conformation of these mannose residues increases the number of possible hydrogen bonds to the surrounding structure, as suggested by *Moorhen*, highlighting the important interactions that will be missed if the modification is modeled incorrectly.

Emerging evidence suggests that tryptophan mannosylation of extracellular proteins, such as those involved in cell guidance functions, could be an important strategy for expanding the functional repertoire of cells expressing these receptors (Akkermans et al., 2022; Jackson et al., 2016; Seiradake et al., 2014). Our analysis pipeline presents a powerful tool to detect these modifications in deposited structural data, and facilitate correct modeling of the relevant structures.

## 3 CONCLUSIONS

*C*‐mannosylation is less well‐known than the more common *N*‐glycosylation. As a result, it is sometimes missed when modeling a new structure. It is also often modeled incorrectly, perhaps in part due to the differences between *C*‐mannose and other glycans, namely, the different ring conformation and linkage. Here, methods to identify and model unmodeled *C*‐mannosylation and to identify and fix incorrectly modeled *C*‐mannosylation are presented.

These methods were used to identify 26 unmodeled mannosylated tryptophans in the PDB across five X‐ray crystal structures and three Cryo‐EM structures. In addition, mannose residues with the wrong linkage or in a high‐energy conformation were identified in 24 models. These residues were removed and remodeled with the correct linkage and ring conformation. The improved models with modeled or remodeled *C*‐mannosylation are available at https://doi.org/10.15124/15288f97-6ef2-4537-a4b4-6ca60944debf.

Across these models, the majority of *C*‐mannosylated tryptophans that were identified to model or remodel were in thrombospondin‐repeat domains (TSR domains). Within these models, the modeling of missing *C*‐mannosylations increased the network of possible hydrogen bonds. In cases where the *C*‐mannosylation was remodeled, the corrected *C*‐mannose residues tended to form denser hydrogen networks with the TSR domains than the original mannose residues in high‐energy conformations and incorrect linkages. This is consistent with the previous investigation of the function of *C*‐mannosylation in stabilizing the TSR domain.

In the future, we hope to integrate the methods presented here for remodeling distorted or incorrect C‐mannosylation into PDB‐REDO so that these improved models are more readily available. In addition, we plan to use this improved dataset of models containing *C*‐mannosylation, and what we have learned regarding commonalities between the *C*‐mannosylated sites, such as being within TSR domains, to identify such sites in AlphaFold models. The same methods presented here could then be used to build the modification into these models.

## 4 METHODS

### 4.1 Identifying distorted *C*‐glycans using *privateer*

As mentioned in the introduction, when not restrained sufficiently, *C*‐mannose residues can be distorted by refinement, ending up in high‐energy conformations or with the wrong linkage to the tryptophan residue. The carbohydrate validation software, *Privateer* (Agirre, Iglesias‐Fernández, et al., 2015), was used to identify these *C*‐mannose residues in glycoprotein structures in the PDB by checking the ring conformation using the Cremer‐Pople puckering coordinates (Cremer & Pople, 1975), glycosidic linkage stereochemistry, and monosaccharide nomenclature to identify potential issues. Any *C*‐glycans flagged as being in high‐energy conformations or as having the wrong linkage were then removed, generating a list of sites to run through the modeling protocol described below.

The functionality to identify incorrectly modeled and distorted *C*‐mannose residues is also available via the *Privateer* Web App (Dialpuri, Bagdonas, Schofield, Pham, Holland, Bond, et al., 2024).

### 4.2 Identifying unmodelled *C*‐glycans

To identify unmodeled *C*‐glycans, tryptophan residues within the consensus sequence (WxxW/C) were identified in protein structures expressed in mammalian cells (John et al., 2021; Krieg et al., 1998). These tryptophan residues are considered potential *C*‐mannosylation sites, searching for any unmodeled density fragments (blobs) nearby. The process for searching for these density blobs follows a different methodology for X‐ray crystal structures and Cryo‐EM structures, as outlined below.

### 4.3 Searching for unmodelled *C*‐mannosylation in X‐ray structures

To detect electron density that could indicate the presence of unmodelled *C‐*mannose residues in crystal structures, the mFo‐DFc map is used to search for positive difference density close to the selected tryptophan residues. Prior to this, the section deletes all hetero‐atoms in the original models using Gemmi (Wojdyr, 2022), which removes any waters or ligands which might be occupying potential *C*‐mannosylation sites. Maps were then recalculated by running 0 cycles of REFMAC5 (Murshudov et al., 2011) with the new model and observed reflection data.

**FIGURE 5:**
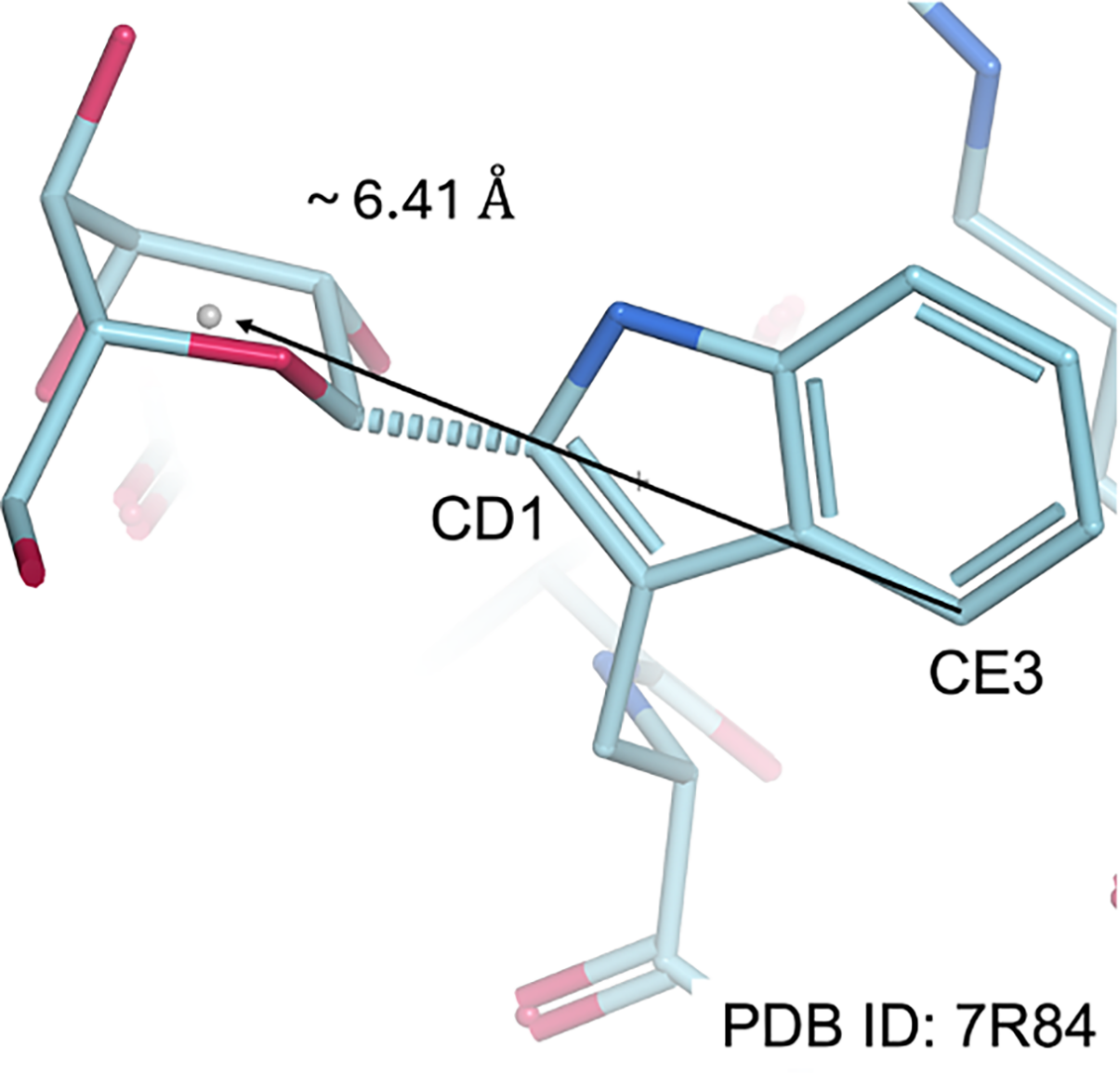
The translation vector from tryptophan to the estimated centroid of *C*‐mannose. A vector from tryptophan (through CE3 to CD1 atoms) is extended 6.41 Å to the estimated mannose centroid (gray dot). The translation distance is the mean distance of 12 *C*‐mannosylation sites in the high‐resolution glycoprotein structures, PDB 7R84 (Wang et al., 2022) and PDB 8CKK (Nagy et al., 2024). The figure illustrates how the translation vector and the estimated centroid are related to the mannose moiety, with *C*‐mannosylation site (chain C, tryptophan 7) in PDB 7R84 (Wang et al., 2022).

In order to find the approximate location of the expected *C*‐mannose residue, a vector of length 6.41 Å is calculated, starting at the CE3 atom of the tryptophan and passing through the CD1 atom. A diagram showing this vector can be seen in Figure 5. The length of the translation vector was calculated as the average distance to the *C*‐mannose centroid across 12 known *C*‐mannosylated tryptophans within two high‐resolution glycoprotein structures with PDB accession codes 7R84 (resolution 1.34 Å; Wang et al., 2022) and 8CKK (resolution 1.56 Å; Nagy et al., 2024). The *C*‐mannose moieties in these two glycoprotein structures were correctly modeled in α‐ᴅ‐mannose and ^1^C_4_ conformation. The recalculated mFo‐DFc map is transformed into a grid using Gemmi (sample rate = 2.0). The average density value of grid points within a 3 Å sphere of the estimated mannose centroid was calculated (Petrescu et al., 2004). Because the numbers of the grid points in the spheres decrease with the low‐resolution models, the methods average the density of the grid point to describe the density of the searching area.

**FIGURE 6:**
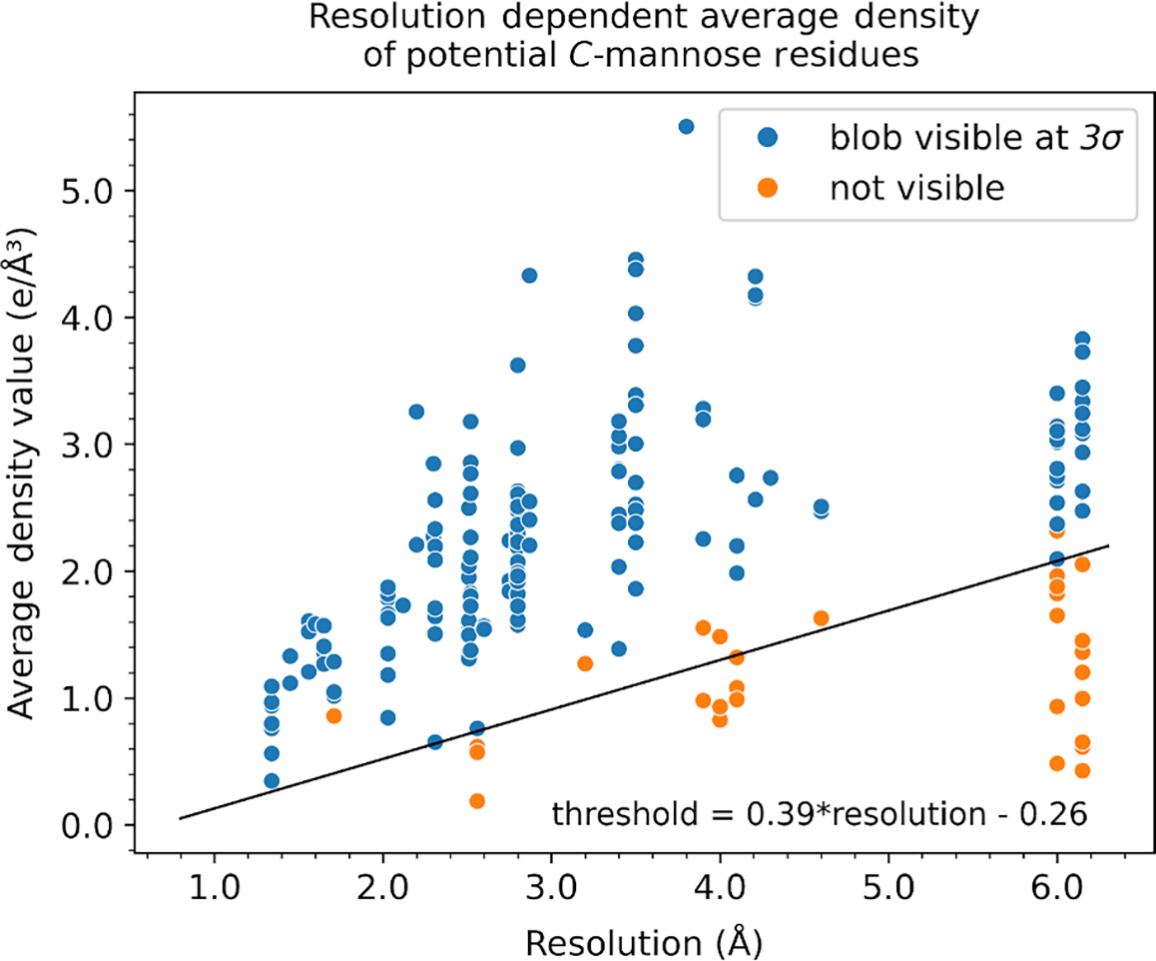
Resolution‐dependent threshold for average density within the sphere for detecting *C*‐mannosylation sites by searching difference density. The difference density search was run on the existing dataset of X‐ray crystal structures containing *C*‐mannosylation deposited in the PDB. The average density of the known *C*‐mannose residues was plotted against resolution. The data points were colored by whether or not there was a positive difference density of mannose residues in the recalculated mFo‐DFc map at *σ* = 3.0 (visible blob or no visible blob at the 3*σ* map contour). The distribution of the *C*‐mannose density in the plot helped to set the scaling thresholds in detecting *C*‐mannosylation in crystal structures.

The method of searching the difference density was tested on the 36 X‐ray crystal structures in the PDB which are known to contain *C*‐mannosylation, providing a dataset of 204 *C*‐mannosylation sites with the *C*‐mannose residues removed. In order to determine the threshold for average density, above which it would be considered likely that a tryptophan was mannosylated, the positive difference density for the known *C*‐mannoses in the recalculated mFo‐DFc maps was checked manually in *Coot* (Emsley & Cowtan, 2004) at the 3*σ* contour level. If there was a positive difference density in line with the translation vector from the tryptophan, the tryptophan was labeled as having the visible blob. The results of this testing are shown in Figure 6, and were used to determine the resolution‐dependent threshold for average density inside the sphere in order to be considered a likely *C*‐mannosylation site. This threshold was found to be Average Density Value≥0.39×Resolution−0.26.

The difference density of 1850 PDB entries was then searched and selected from a mirror of the PDB (downloaded on July 25, 2024), based on the fact that their protein chains were expressed in mammalian cells and that they contained the WxxW|C consensus sequence. If the mean value of density within the sphere at the determined location was above the resolution‐dependent threshold, the corresponding tryptophan residue was considered a potential *C*‐mannosylation site.

### 4.4 Searching for unmodeled *C*‐mannosylation in Cryo‐EM structures

In Cryo‐EM structures, the primary EM map of the original structure was used to detect unmodeled *C‐*mannose. The same translation vector from tryptophan to the estimated mannose centroid (distance = 6.411 Å) was used. The method searches in a cube of edge 7 Å around this centroid, across a grid of points 1 Å apart, interpolating the density map onto these points. The sum of the density within the volume of this cube is then calculated, with no averaging required, as the number of grid points within the cube is not dependent on resolution and does not change across models.

The six Cryo‐EM structures deposited in the PDB which contain *C*‐mannosylation were used to test the method and determine the threshold for the sum of the density within the cube volume. These 6 models contained 49 *C*‐mannosylation sites. Across these sites, the minimum density sum found when searching the primary was 308.65 V. This was chosen as the threshold, above which the neighboring tryptophan would be considered to be a potential *C*‐mannosylation site.

The density map of 4647 Cryo‐EM structures was then searched and selected from a mirror of the PDB (downloaded on July 25, 2024), based on the fact that their protein chains were expressed in mammalian cells and that they contained the WxxW|C consensus sequence. If the sum of the density within the cube at the determined location was above the threshold, the corresponding tryptophan residue was considered a potential *C*‐mannosylation site.

### 4.5 *C*‐mannosylation modeling protocol

The list of sites generated by the search for incorrect or unmodeled *C*‐glycans is used as input, modeling an α‐ᴅ‐mannose residue in the correct ^1^C_4_ chair conformation covalently bonded to the identified tryptophan.

In order to identify the ideal torsion and bond angles at which the α‐ᴅ‐mannose should be bonded to the tryptophan residue, an analysis of the currently deposited glycoprotein structures including *C*‐mannosylation was carried out. The *Privateer Database* (Dialpuri, Bagdonas, Schofield, Pham, Holland, & Agirre, 2024) was used to identify glycoprotein structures in the PDB which included *C*‐mannosylated tryptophan. The diagnostic information included in the database was then used to identify which of these models had no potential issues in these glycans. These models then formed the dataset to analyze.

**FIGURE 7:**
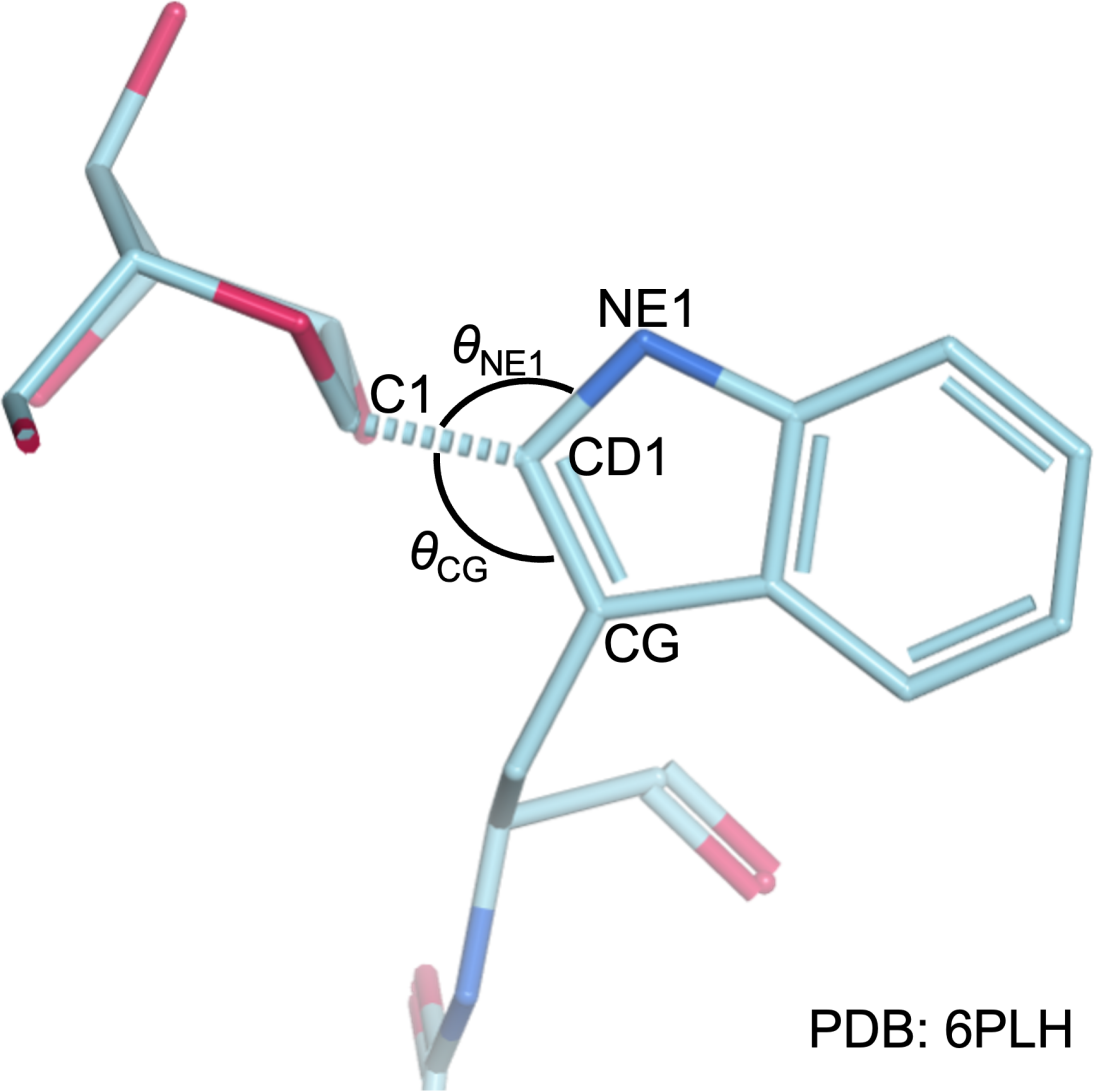
Definitions of the bond angles used to approximate position, the mannose when bonding it to a tryptophan. *θ*_CG_ defines the angle between the glycosidic linkage and the CD1—CG bond, and *θ*_NE1_ defines the angle between the glycosidic linkage and the CD1—NE1 bond. Not shown is *θ*_plane_, the angle between the glycosidic linkage and the vector normal to the plane of the aromatic ring of the tryptophan.

The bond angles of the linkage were only considered for two high‐resolution glycoprotein structures: 7R84 at 1.34 Å resolution (Wang et al., 2022) and 6PLH at 1.6 Å resolution (John et al., 2021), resulting in a sample size of *N* = 9. These glycoprotein structures were chosen as resolving the bond angle accurately enough was not possible in poorer‐resolution models. Definitions of the bond angles referred to in the text are given in Figure 7. From the two high‐resolution models, the average bond angles were found to be *θ*_CG_ = 131 ± 3, *θ*_NE1_ = 118 ± 3, and *θ*_plane_ = 90 ± 5. These are the angles that were used to set the approximate location of the mannose residue once its O1 atom had been overlaid with the CD1 atom of the tryptophan, before further fine‐tuning occurred via the torsion angles.

**FIGURE 8:**
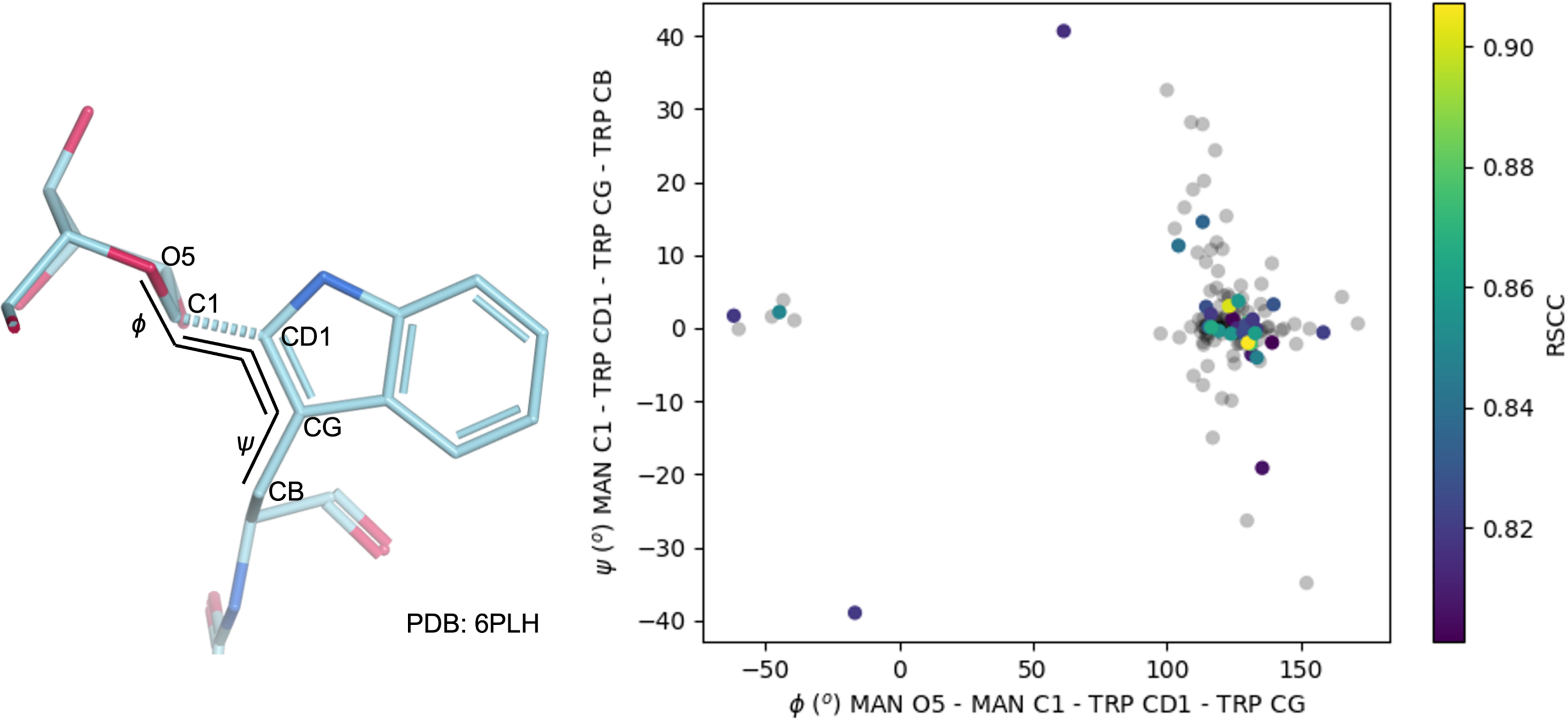
Distribution of torsion angles *ɸ* (MAN‐O5—MAN‐C1—TRP‐CD1—TRP‐CG) and *ѱ* (MAN‐C1—TRP‐CD1—TRP‐CG—TRP‐CB) defined in the left panel within *C*‐mannosylated protein structures in the PDB. The color of the points in the plot represents the RSCC of the mannose residue with any point where RSCC <0.8 grayed out. To minimize the impact of outliers, the median of each angle was calculated using the high‐quality RSCC data along with the standard deviation, giving *ɸ* = 130 ± 50 and *ѱ* = 0 ± 10. Figure produced using https://moorhen.org/.

The average torsion angles of the glycosidic linkage were calculated across the full dataset of *C*‐glycans with no conformation or anomer issues and with RSCC >0.8, totaling a sample size of *N* = 31. The median average was chosen to avoid outliers skewing the result. Outliers with high RSCC, indicating a good fit to the density, can be caused by interactions with the surrounding structure forcing the torsion angles of the glycosidic linkage away from the ideal. The angle definitions and dataset analysis are shown in Figure 8, resulting in median torsion angles of *ɸ* = 130 ± 50° and *ѱ* = 0 ± 10°. These values along with their standard deviations were used to set the ideal torsion angles when modeling the mannose residue bonded to the tryptophan, and the standard deviations were used to set the range of different torsion angles that the structure was rotated through in order to minimize clashes. The median torsion angles found are within one standard deviation of those given by AceDRG. This torsion angle analysis for the TRP‐MAN carbon–carbon glycosidic linkage will also be added to the existing torsion database within *Privateer* (Dialpuri et al., 2023), to better aid in validating models containing C‐mannosylation in the future.

A high‐resolution α‐ᴅ‐mannose residue in the correct ^1^C_4_ ring conformation was then taken from an X‐ray crystal structure of mouse BAI1 (ADGRB1) TSR3 domain in the P21 space group with PDB accession code 7R84 (Wang et al., 2022). This α‐ᴅ‐mannose residue was then placed into the identified sites. This was done by overlaying the O1 atom of the α‐ᴅ‐mannose with the CD1 atom of the tryptophan residue, rotating the α‐ᴅ‐mannose until the correct bond angles were achieved, then rotating it again to achieve the ideal torsion angles. If the α‐ᴅ‐mannose was found to have any clashes with other nearby residues, it was rotated further within bounds determined by the above torsion angle analysis in order to minimize clashes. If all the clashes could not be eliminated, it was determined that there was not enough space for the α‐ᴅ‐mannose at that location, and the sugar was deleted from the 3D model. The functionality to perform this process was written into *Privateer* primarily in C++ along with wrapper functions in Python.

Refinement of the 3D models was then carried out using *Servalcat*/*REFMAC5* (Murshudov et al., 2011; Yamashita et al., 2023) using restraint files for the glycans present generated using *Privateer* (Atanasova et al., 2022). The option to generate these restraints for refinement in Phenix was also added to the code. *Privateer* was then used to calculate the RSCC of the newly modeled glycans in order to determine the fit to the experimental data. When trying to identify unmodeled *C*‐glycans, any modeled mannose with an RSCC <0.5, as calculated using the refined map, was deemed to be poorly supported by the experimental data and so was automatically deleted. This threshold was chosen as it has previously been deemed to be a low value indicating poor fit to the density (Agirre, Davies, et al., 2015). Any mannose residues with an RSCC >0.5 were inspected manually to determine the confidence in the modeled *C‐*mannosylation. This threshold was determined by considering the dataset of existing 3D models containing *C*‐mannosylation.

In addition to using RSCC, the (re)modeled *C*‐mannose residues in Cryo‐EM structures were validated with *Q*‐score by running MapQ (Pintilie et al., 2020) in Chimera 1.13 (Pettersen et al., 2004). To calculate the *Q*‐score for all atoms in the chain, the default setting of sigma = 0.6 was used.

**FIGURE 9:**
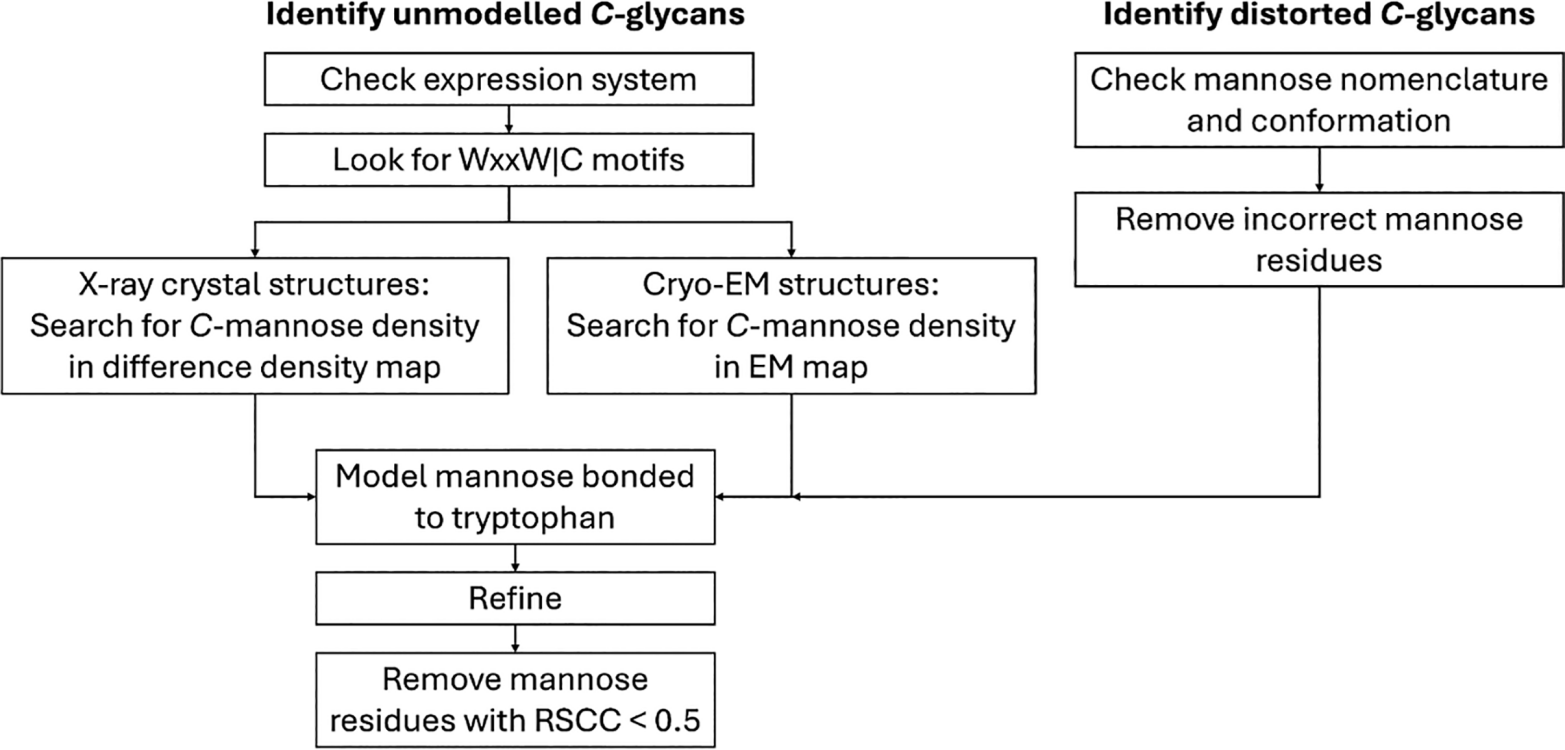
Diagram of methodology to identify and model unmodeled *C*‐mannosylation and to remodel incorrect *C*‐mannosylation. To identify unmodeled *C*‐mannosylation sites, first, the expression system of the protein chain was checked, with chains expressed in metazoan cells passing on to the next step. Then, the chain was searched to see if it contained the consensus sequence for *C*‐mannosylation (WxxW|C). If a consensus sequence was found, in X‐ray crystal structures, the difference density close to that tryptophan was then searched for a blob of positive difference density. In Cryo‐EM structures, the density map close to the tryptophan was searched for a blob of density. An ɑ‐ᴅ‐mannose residue in the ^1^C_4_ conformation was then modeled at those tryptophan residues. Then, the models were refined using Refmac5 (for X‐ray structures) or Servalcat (for Cryo‐EM structures), with restraint dictionaries for the glycans generated using *Privateer*. Any mannose residues with an RSCC <0.5 were then automatically removed. To identify *C*‐mannose residues that have been incorrectly modeled, *Privateer* was used. Any which were flagged as containing a conformation issue or as being the wrong anomer were removed from the structure, and remodeled using the same protocol for modeling unmodeled *C*‐glycans.

The process was automated via Python and is summarized in Figure 9. The output of the code is a PDB file and an mmCIF file containing the new structure with either fixed or additionally modeled *C*‐glycans, along with a CSV file summarizing the glycans which have been changed or added.

## AUTHOR CONTRIBUTIONS

**Lou Holland:** Software; methodology; writing – original draft. **Phuong Thao Pham:** Conceptualization; methodology; software; writing – original draft. **Haroldas Bagdonas:** Software. **Jordan S. Dialpuri:** Conceptualization; methodology; software; data curation; writing – review and editing; investigation; writing – original draft; supervision. **Lucy C. Schofield:** Writing – review and editing; data curation; visualization; writing – original draft. **Jon Agirre:** Supervision; conceptualization; investigation; funding acquisition; software; validation; resources.

## FUNDING INFORMATION

Lou Holland is funded by The Royal Society (URF\R\221006). Phuong Thao Pham is a self‐funded PhD student. Haroldas Bagdonas was funded by The Royal Society (grant No. RGF/R1/181006). Jordan Dialpuri is funded by the Biotechnology and Biological Sciences Research Council (BBSRC; grant No. BB/T0072221). Lucy Schofield is funded by STFC/CCP4 PhD studentship agreement 4462290 (York)/S2 2024 012 (STFC) awarded to Jon Agirre. Jon Agirre is a Royal Society University Research Fellow (awards UF160039 and URF\R\221006) and is partly funded by the BBSRC (grant no. BB/Y00888X/1).

## Supporting information


Data S1.

## Data Availability

The full source code implemented and used here is available at https://github.com/glycojones/privateer and will be released in binary form via an update to the CCP4 suite (Agirre et al., 2023). The data used are publicly available via the PDB. The improved structures which were output by the code are available at https://doi.org/10.15124/15288f97-6ef2-4537-a4b4-6ca60944debf.

## 1

A summary of the glycoprotein structures found to have unmodelled *C*‐mannosylation is given in Table A1. A summary of the glycoprotein structures where distorted or incorrect *C*‐mannose residues were improved is given in Table A2 for X‐ray crystal structures, and in Table A3 for Cryo‐EM structures.

**TABLE A1:** Potential *C*‐mannosylation sites where the PTM was previously unmodelled.

Method	PDB code	Resolution (Å)	Residue	UniProt	RSCC	*Q*‐score
X‐ray diffraction	4v2a	2.40	A/245	Q6ZN44	0.77	
			A/248	Q6ZN44	0.75	
	5ftt	3.40	A/256	F1LW30	0.62	
			E/253	F1LW30	0.93	
			E/256	F1LW30	0.84	
	7noz	3.90	D/385	P27918	0.60	
	7za2	4.60	E/256	F1LW30	0.69	
	7za3	4.00	E/256	F1LW30	0.91	
			G/256	F1LW30	0.84	
			H/256	F1LW30	0.84	
Cryo‐EM	8b0f	3.00	B/29	P01031	0.64	0.63
			B/32	P01031	0.60	0.59
			D/497	P07358	0.72	0.63
			E/512	P07357	0.59	0.51
			E/515	P07357	0.67	0.48
	8b0h	3.30	B/568	P13671	0.59	0.54
			B/571	P13671	0.56	0.53
			D/497	P07358	0.61	0.57
			E/14	P07357	0.59	0.56
			E/512	P07357	0.62	0.61
			E/515	P07357	0.55	0.56
	8b0g	3.30	B/568	P13671	0.53	0.41
			B/571	P13671	0.61	0.54
			D/497	P07358	0.71	0.58
			E/512	P07357	0.69	0.58
			E/515	P07357	0.68	0.58

*Note*: The resolution of each structure is given, along with the PDB assigned chain and residue ID of the tryptophan, the corresponding UniProt ID, the RSCC, and *Q*‐scores of the modeled mannose.

**TABLE A2:** Fixed *C*‐mannosylation sites where the PTM was previously incorrectly modeled in X‐ray crystallography structures.

PDB code	Residue	Problem	Original RSCC	Fixed RSCC	Original H‐bonds	Fixed H‐bonds	Notes
3ojy	A/14	High energy conformation (3s1) Anomer issue (BMA)	0.74	0.82	4	4	
	A/512	High energy conformation (3s1) Anomer issue (BMA)	0.68	0.78	3	3	
	A/515	High energy conformation (3s1) Anomer issue (BMA)	0.75	0.80	3	4	
	A/518	High energy conformation (Oh1) Anomer issue (BMA)	0.59	0.47	–	–	Removed
	B/16	High energy conformation (3Ob) Anomer issue (BMA)	0.76	0.79	2	3	
	B/19	High energy conformation (4c1) Anomer issue (BMA)	0.55	0.43	–	–	Removed
	B/497	High energy conformation (3s1) Anomer issue (BMA)	0.86	0.85	3	3	
	B/500	High energy conformation (3Ob) Anomer issue (BMA)	0.49	0.50	–	–	Removed
3t5o	A/8	High energy conformation (1s3)	0.92	0.89	3	3	
	A/11	High energy conformation (4c1)	0.83	0.77	2	5	
	A/547	High energy conformation (4c1)	0.82	0.81	3	4	
	A/550	High energy conformation (2h1)	0.68	0.81	3	2	
3tgx	A/195	High energy conformation (2h1) Anomer issue (BMA)	0.80	0.74	3	4	
	C/195	High energy conformation (2h1) Anomer issue (BMA)	0.72	0.86	5	6	
	E/195	High energy conformation (2h1) Anomer issue (BMA)	0.78	0.80	4	4	
	G/195	High energy conformation (2h1) Anomer issue (BMA)	0.73	0.88	5	7	
	I/195	High energy conformation (2h1) Anomer issue (BMA)	0.80	0.85	4	4	
	K/195	High energy conformation (2h1)	0.79	0.85	4	4	
	M/195	High energy conformation (2h1)	0.78	0.84	5	5	
	O/195	High energy conformation (2h1) Anomer issue (BMA)	0.70	0.77	2	5	
3vn4	A/387	High energy conformation (3s1) Anomer issue (BMA)	0.72	0.67	3	4	
4e0s	B/8	High energy conformation (4c1)	0.84	0.89	2	4	
	B/11	High energy conformation (4c1)	0.59	0.86	2	4	
	B/547	High energy conformation (4c1)	0.83	0.90	3	4	
	B/550	High energy conformation (ev1)	0.88	0.92	3	3	
4nzd	A/195	High energy conformation (3Ob) Anomer issue (BMA)	0.84	0.86	5	6	
	B/195	High energy conformation (3s1) Anomer issue (BMA)	0.84	0.85	2	4	
	C/195	High energy conformation (3Ob) Anomer issue (BMA)	0.85	0.85	4	6	
5lfu	A/22	High energy conformation (ev2)	0.47	0.67	1	1	
5m5e	B/194	High energy conformation (3h2)	0.87	0.89	5	5	
6cxo	A/26	High energy conformation (3s1) Anomer issue (BMA)	0.88	0.83	3	3	
	B/26	High energy conformation (3s1) Anomer issue (BMA)	0.86	0.89	2	3	
6rus	A/83	High energy conformation (3ev)	0.62	0.77	3	3	
	B/263	High energy conformation (ev4)	0.48	0.66	2	3	
6s08	A/388	High energy conformation (14b)	0.75	0.62	3	2	
6s0a	A/382	High energy conformation (b3O)	0.82	0.77	3	3	
6s0b	A/382	High energy conformation (4c1)	0.73	0.67	1	5	
	A/385	High energy conformation (1h2)	0.65	0.73	3	3	
7b26	B/388	High energy conformation (1s5)	0.83	0.86	3	3	
7noz	D/382	High energy conformation (1h2)	−0.30	0.66	3	1	
7r85	A/10	High energy conformation (1s5)	0.68	0.46	–	–	Removed
7za2	F/256	Anomer issue (BMA)	0.48	0.18	–	–	Removed
	G/253	Anomer issue (BMA)	0.20	0.20	–	–	Removed
7za3	F/256	Anomer issue (BMA)	−0.11	0.79	0	2	
	G/253	Anomer issue (BMA)	−0.17	0.28	–	–	Removed

*Note*: The location of the tryptophan in the structure is given via the PDB chain ID and the PDB residue ID. The problem in the original structure is summarized, along with the original RSCC of the mannose residue to be compared with the RSCC of the mannose residue after fixing and refining. The number of H‐bonds suggested by Moorhen between the mannose residue and the rest of the structure (excluding H‐bonds that are internal within the mannose residue or are formed with nearby water molecules) is also given for the original and fixed models. As this number represents all possible hydrogen bonds regardless of the orientation of the hydrogen atoms, it is likely to be an overestimate. Some of the mannose residues were removed after fixing, as they were deemed to be poorly supported by the experimental data. In these cases, the fixed RSCC given is the RSCC of the mannose residue after fixing and refining, before it was removed. All of the removed cases had an RSCC <0.6 prior to removal.

**TABLE A3:** Fixed *C*‐mannosylation sites where the PTM was previously incorrectly modeled in Cryo‐EM structures.

PDB code	Residue	Problem	Original RSCC	Fixed RSCC	Original *Q*‐score	Fixed *Q*‐score
6dlw	A/27	High energy conformation (4c1) Anomer issue (BMA)	0.41	0.50	0.53	0.50
	B/27	High energy conformation (4c1) Anomer issue (BMA)	0.32	0.49	0.53	0.50
	C/27	High energy conformation (4c1) Anomer issue (BMA)	0.33	0.44	0.51	0.51
	D/27	High energy conformation (4c1) Anomer issue (BMA)	0.40	0.42	0.51	0.54
	E/27	High energy conformation (4c1) Anomer issue (BMA)	0.43	0.46	0.51	0.53
	F/27	High energy conformation (4c1) Anomer issue (BMA)	0.37	0.42	0.50	0.53
	G/27	High energy conformation (4c1) Anomer issue (BMA)	0.38	0.46	0.53	0.53
	H/27	High energy conformation (4c1) Anomer issue (BMA)	0.41	0.44	0.52	0.55
	I/27	High energy conformation (4c1) Anomer issue (BMA)	0.38	0.40	0.53	0.53
	J/27	High energy conformation (4c1) Anomer issue (BMA)	0.38	0.43	0.51	0.54
	K/27	High energy conformation (4c1) Anomer issue (BMA)	0.38	0.43	0.51	0.53
	L/27	High energy conformation (4c1) Anomer issue (BMA)	0.42	0.50	0.53	0.53
	M/27	High energy conformation (4c1) Anomer issue (BMA)	0.38	0.46	0.54	0.56
	N/27	High energy conformation (4c1) Anomer issue (BMA)	0.42	0.38	0.51	0.57
	O/27	High energy conformation (4c1) Anomer issue (BMA)	0.42	0.44	0.51	0.55
	P/27	High energy conformation (4c1) Anomer issue (BMA)	0.39	0.41	0.52	0.55
	Q/27	High energy conformation (4c1) Anomer issue (BMA)	0.42	0.44	0.52	0.55
	R/27	High energy conformation (4c1) Anomer issue (BMA)	0.41	0.44	0.51	0.53
	S/27	High energy conformation (4c1) Anomer issue (BMA)	0.38	0.42	0.50	0.54
	T/27	High energy conformation (4c1) Anomer issue (BMA)	0.38	0.45	0.51	0.52
	U/27	High energy conformation (4c1) Anomer issue (BMA)	0.36	0.45	0.49	0.51
	V/27	High energy conformation (4c1) Anomer issue (BMA)	0.36	0.43	0.50	0.52
7nyc	C/14	High energy conformation (4c1) Anomer issue (BMA)	−0.16	0.07	0.12	0.08
	D/16	High energy conformation (Os2) Anomer issue (BMA)	0.16	0.14	0.42	0.27
	D/497	High energy conformation (1s5) Anomer issue (BMA)	−0.15	−0.05	0.44	−0.09
	E/14	High energy conformation (Os2) Anomer issue (BMA)	0.42	0.57	0.45	0.38
	E/512	High energy conformation (1hO) Anomer issue (BMA)	0.09	0.32	0.28	0.17
	E/515	High energy conformation (b25) Anomer issue (BMA)	−0.14	0.34	0.16	0.21
	G/27	High energy conformation (4c1) Anomer issue (BMA)	0.2q	0.69	0.57	0.15
7nyd	C/484	High energy conformation (4ev) Anomer issue (BMA)	0.13	0.09	0.23	−0.03
	D/19	Anomer issue (BMA)	−0.07	0.01	0.13	0.24
	D/500	High energy conformation (1s5) Anomer issue (BMA)	0.14	0.27	0.23	0.47
	E/14	High energy conformation (4c1) Anomer issue (BMA)	0.19	0.58	0.29	0.33
	E/512	High energy conformation (b25) Anomer issue (BMA)	−0.02	0.58	0.46	0.23
	G/27	High energy conformation (Oev) Anomer issue (BMA)	0.03	0.34	0.15	0.19
8de6	A/27	High energy conformation (4c1) Anomer issue (BMA)	0.24	0.38	0.51	0.45
	A/30	High energy conformation (4c1) Anomer issue (BMA)	0.45	0.36	0.62	0.52
	C/27	High energy conformation (4c1) Anomer issue (BMA)	0.39	0.58	0.57	0.62
	C/30	High energy conformation (4c1) Anomer issue (BMA)	0.27	0.23	0.44	0.54
	G/27	High energy conformation (4c1) Anomer issue (BMA)	0.36	0.61	0.50	0.57
	G/30	High energy conformation (4c1) Anomer issue (BMA)	0.26	0.29	0.39	0.37
8 g04	B/269	High energy conformation (4c1)	0.35	0.58	0.47	0.57
	B/272	High energy conformation (4c1)	0.22	0.35	0.38	0.42
	B/474	High energy conformation (4c1)	−0.23	0.05	0.47	−0.03
	C/269	High energy conformation (4c1)	0.41	0.70	0.50	0.56
	C/272	High energy conformation (4c1)	0.08	0.13	0.28	0.29
	C/474	High energy conformation (4c1)	0.13	0.16	0.02	0.26
8u18	A/465	High energy conformation (4c1)	0.23	0.23	0.27	0.37
	B/465	High energy conformation (4c1)	0.13	0.14	0.16	0.12

*Note*: The location of the tryptophan in the structure is given via the PDB chain ID and the PDB residue ID. The problem in the original structure is summarized, along with the original RSCC and *Q*‐scores of the mannose residue to be compared with the RSCC and *Q*‐scores of the mannose residue after fixing and refining. Unlike the X‐ray crystal structures, mannose residues with poor RSCC were not removed, as in many cases, it appeared the problem may stem from issues in the surrounding structure. As such, no hydrogen bond analysis was carried out.
