# Supplementary material for: Methods for detecting, building, and improving tryptophan mannosylation in glycoprotein structures
Source: Protein Sci. 2025 Jan 22;34(2):e70025. doi: 10.1002/pro.70025 (PMC11751905; doi:10.1002/pro.70025)
Supplement: Supplementary file 1 — Data S1. [file PRO-34-e70025-s001.docx]

## Supplementary Material

An additional example of a distorted *C*-mannose residue that was fixed in complement component polyC9 is shown in Figure S1. This was found in the Cryo-EM structure with PDB accession code 6DLW.


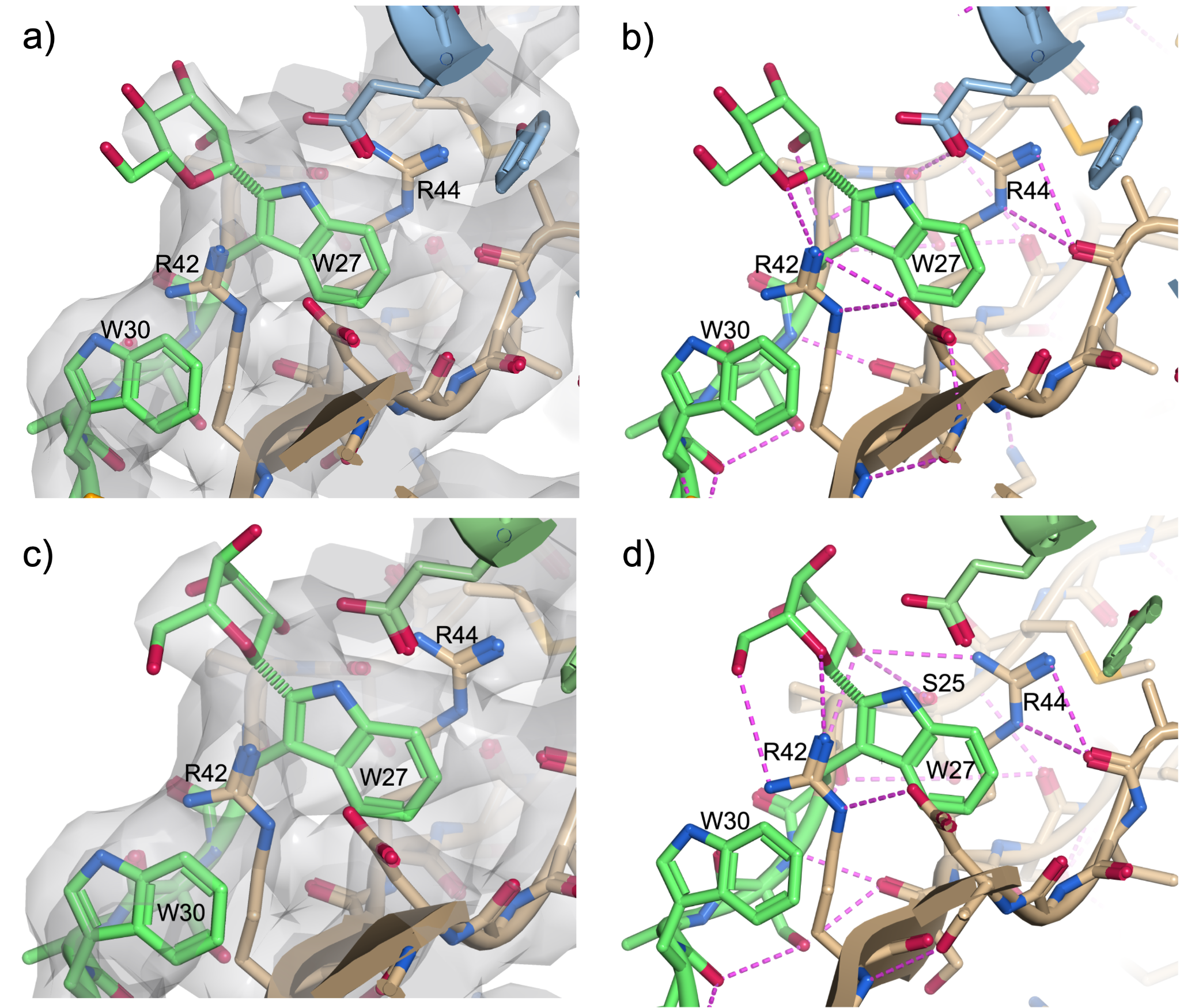


**Figure S1.** An example of using the methods presented here to fix a distorted *C*-mannose residue in a Cryo-EM structure with PDB accession code 6DLW. Panels (a) and (b) show the original structure while panels (c) and (d) show the fixed structure. The mannose residue in question is bonded to TRP-27 in chain A which sits in the core of a TSR domain, with two tryptophan residues forming an alternating ladder like structure with two arginine residues. In the original structure, the mannose residue was beta-linked to the tryptophan and was in the high energy ^4^C_1_ conformation. It was fixed so that it was alpha-linked and in the lowest energy ^1^C_4_ conformation. In the original structure, moorhen suggested two possible hydrogen bonds between the mannose residue and nearby residues, whereas in the fixed structure, moorhen suggested five possible hydrogen bonds. This figure was produced using <https://moorhen.org/>.

A plot showing the sum of the density in the cube (as described in the section titled “Searching Density Map for Cryo-EM Structures”) for the existing *C*-mannosylated Cryo-EM structures. Due to the small number of points, a resolution dependent threshold could not be reliably found, so instead a constant cut-off was chosen corresponding to the lowest value in the dataset. This is shown in Figure S2.


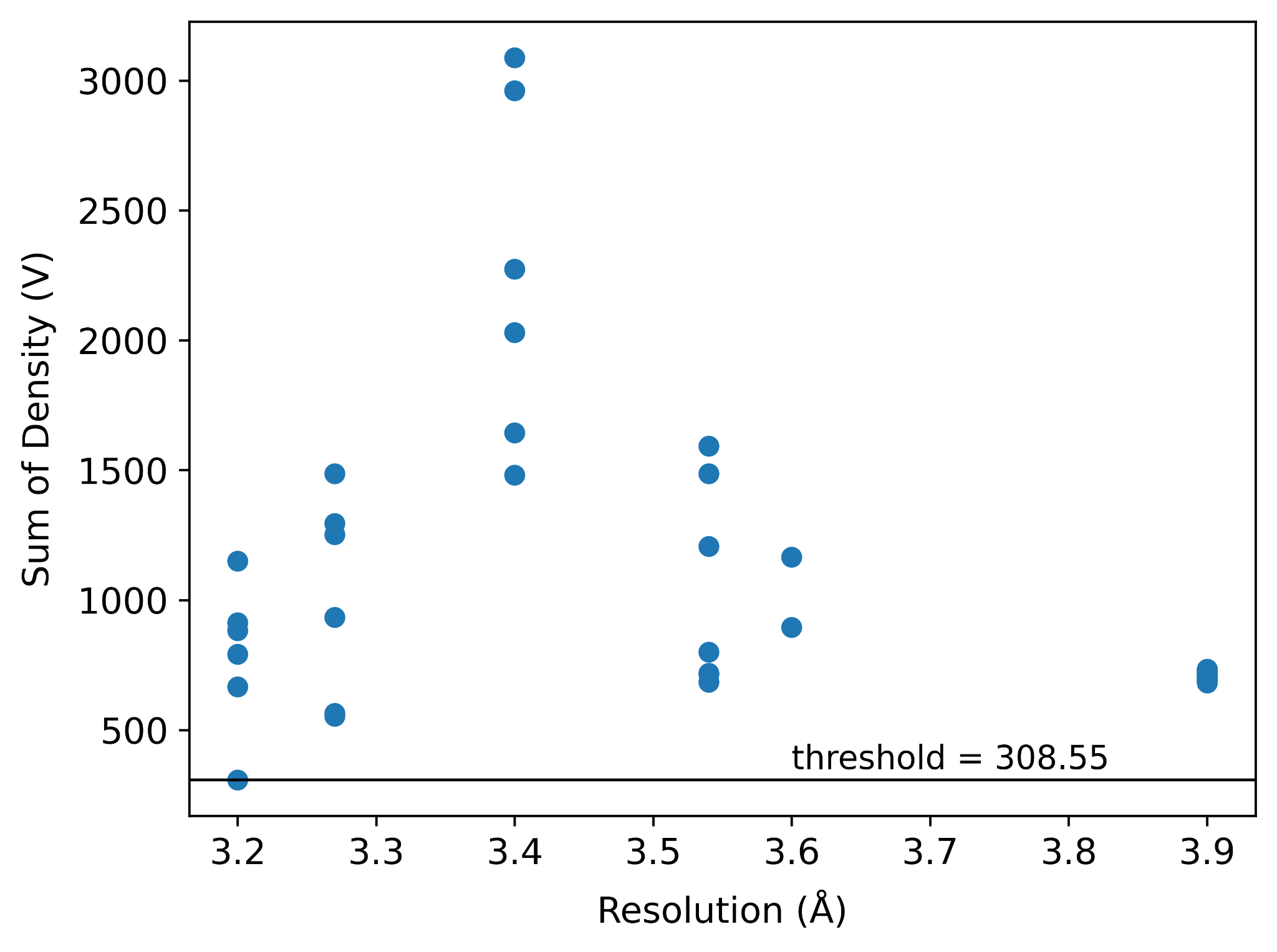


**Figure S2:** Threshold in detecting *C*-mannosylation of map search. The built algorithm of map search was run on the deposited *C*-mannosylation Cryo-EM structures. The sum density of the known *C*-mannosylated tryptophans (blue dots) were plotted against the resolutions of their models, and the minimal value of the sum density was set as the threshold in detecting unmodelled *C*-mannosylation in Cryo-EM structures.
